# Urban Life as Risk Factor for Aspergillosis

**DOI:** 10.3389/fcimb.2020.601834

**Published:** 2020-11-02

**Authors:** Claudia Grehn, Patience Eschenhagen, Svenja Temming, Uta Düesberg, Konrad Neumann, Carsten Schwarz

**Affiliations:** ^1^ Department of Pediatric Pulmonology, Immunology and Intensive Care Medicine, CF Center, Charité—Universitätsmedizin Berlin, Berlin, Germany; ^2^ Mukoviszidose Institut, Bonn, Germany; ^3^ Institute for Biometry and Clinical Epidemiology, Charité—Universitätsmedizin Berlin, Berlin, Germany

**Keywords:** cystic fibrosis, aspergillosis, *Aspergillus fumigatus*, allergic bronchopulmonary aspergillosis, urban life, rural life, respiratory infection

## Abstract

*Aspergillus fumigatus* (*Af*) frequently colonizes the airways of patients with cystic fibrosis (CF) and can cause severe diseases, such as allergic bronchopulmonary aspergillosis, *Af* bronchitis or even *Af* pneumonia. However, risk factors, including environmental factors, for acquiring *Af* in the respiratory tract of patients with CF are rarely studied and described. The aim of this study was to investigate whether urban or rural life could affect colonization with *Af* in the respiratory tract of patients with CF. Due to privacy policy, registry data are usually not linked to patients´ home addresses. It is therefore very difficult to analyze the influence of the patient´s residential environment. This prospective questionnaire survey was carried out in 31 German CF centers in 2018. Only completed surveys, including a clearly assigned type of residential area were included. Statistical analysis was performed by chi-squared test and logistic regression models. A total of 1016 questionnaires were analyzed (Patients` age: 23 ± 13; 0–88 years; female gender: n=492; 48%). The majority of patients with CF live in large cities (n =314; 30.9%) or urban districts (n=461; 45.4%). Prevalence of 30.2% was found for *Af*, within the 12 months of investigation period. *Af* colonization was significantly associated with urban life (p=0.004). Urban live should be considered as possible new risk factor for colonization with *Af* in the respiratory tract of patients with CF. These new results may raise the awareness of the influence of environmental factors on patient outcomes and should be included in patient guidance and preventive measures.

## Introduction

The life-limiting autosomal recessive disorder Cystic fibrosis (CF) is characterized by abnormal viscous secretions in the lower airways leading to obstruction, inflammation, tissue damage and destruction of the lung (Elborn, 2016).In patients with CF, fungal colonization of the respiratory tract is frequently found (Pihet et al., 2009). *Aspergillus fumigatus* (*Af*), the most common filamentous fungus in CF patients with a prevalence of 30 %, is becoming more frequently isolated (Ziesing et al., 2016; Brandt et al., 2018; Schwarz et al., 2018; Hong et al., 2020). The airways are constantly exposed to fungal spores, yet which are only a part of the airway pathogens in the environment. Hundreds of *Aspergillus* spores are inhaled every day, and due to their small size, they directly enter the small airways (Latge, 1999). In CF airways, these spores are able to persist and germinate, leading to immune responses with leukocyte infiltration and mucus accumulation (Singh et al., 2018). *Aspergillus* species are associated with negative patient outcome and can cause severe diseases, like allergic bronchopulmonary aspergillosis (ABPA) (Singh et al., 2018). About 1–15 % of the CF patients suffer from this serious *Aspergillus*-related disease (Stevens et al., 2003). ABPA is caused by hypersensitivity to allergens of *Af* (Janahi et al., 2017), is characterized by a variety of clinical and immunological responses (Stevens et al., 2003) and is associated with a negative effect on lung function (Kraemer et al., 2006). Until now, diagnosis of ABPA in patients with CF remains challenging (Janahi et al., 2017). In future, approximately 70 % of the world´s population will live in urban areas and modern urbanites will spend approximately 90 % of their times indoors (Rydin et al., 2012; Leung and Lee, 2016; Flies et al., 2019). If the patient’s environment poses a potential risk to *Aspergillus* colonization, preventive measures could be identified. Limited work has been done examining *Af* colonization in CF patients regarding their type of residential area in Germany. As registry data of the patient are not linked with their home address, this questionnaire survey was conducted to gain evidence of an association between aspergillosis and urban or rural life.

## Materials and Methods

### Design and Development

This questionnaire survey was conducted in German CF centers in 2018. The target population was people with CF independent of age and living in Germany. Participation was voluntary. Ethical aspects were considered and approval for the study was gained by the Ethics Committee of the Charité – Universitätsmedizin Berlin (EA2/057/18).

The questionnaire included several items, which are not documented in the medical data base German Cystic Fibrosis Registry. Therefore, beside patients’ age and sex and their history of *Af* colonization and ABPA diagnosis within the last 12 months, the postal code of the place of residence, were asked for in the questionnaire. For a positive of *Af* colonization within the 12 month of observation period, at least one positive microbiological indication was required. The ABPA diagnosis was determined in every single CF center and was based on the minimal 2003 Cystic Fibrosis Foundation (CFF) consensus criteria (Stevens et al., 2003). No other *Af* associated phenotypes were considered. Besides *Af*, no other *Aspergillus* spp. were included. The questionnaire was sent to 94 CF centers in Germany in 2018 and data of 1,477 patients have been received from 31 CF centers. Postal code was correlated to rural and urban area with data from the German Federal Institute for Building, Urban and Spatial Research (BBSPR). The population density was correlated from the postal code with data from the German Statistic Federal Office (DESTATIS).

### Statistical Analysis

Only completed questionnaires with distinct classification for the type of residential area and population density were included in final analysis. The residential area was classified as large city, urban district, rural district and sparsely populated rural area. Population density was graduated in high, medium and low. Means and standard deviations were calculated for metrical variables. Frequency and percentage were used for categorical variables. Associations of categorical variables were analyzed using Pearson´s chi-square tests. In addition, all parameters were considered within binary logistic regression analysis. For multiple binary logistic regression analysis (only for *Af* colonization), the following parameters have been adjusted: age (years) and residential area (large city, urban district, rural district and sparsely populated rural area). In this exploratory study the level of significance (α=0.05) was not adjusted for multiple testing. Statistics were calculated using IBM SPSS Statistics 24.

## Results

A total of 1,465 patients with CF participated and a total of 1,016 completed questionnaires were analyzed. Of the included patients, 48.4% were female (n = 492; male: n = 524; 51.6 %). The mean age was 23 ± 13 years (range: 0–88 years). The majority of CF patients live in large cities (n = 314; 30.9 %) and urban districts (n = 461; 45.4 %; **Table 1**).

**Table 1:** Demographic baseline characteristics.

	All CF patients	CF patients < 18 years	CF patients ≥ 18 years	P value
	1016 (100 %)	395 (38.9 %)	621 (61.1%)	
Female sex, n (%)	492 (48.4%)	197 (49.9%)	295 (47.5%)	0.461
Age (years), mean ± SD (range)	23 ± 13 (0 - 88)	10 ± 5 (0–17)	31 ± 10 (18–88)	
*Af* colonization^a^, n (%)	307 (30.2%)	73 (18.5%)	234 (37.7%)	<0.001
ABPA^a^, n (%)	165 (16.2%)	30 (7.6%)	135 (21.7%)	<0.001
Type of residential area, n (%)				
Large city	314 (30.9 %)	91 (23.0%)	223 (35.9%)	<0.001
Urban district	461 (45.4 %)	186 (47.1%)	275 (44.3%)	0.401
Rural district	141 (13.9 %)	68 (17.2%)	73 (11.8%)	0.016
Sparsely populated rural area	100 (9.8 %)	50 (12.7%)	50 (8.0%)	0.018
Population density, n (%)				
High	137 (13.5%)	46 (11.6%)	91 (14.7%)	0.188
Medium	601 (59.2%)	213 (53.9%)	388 (62.5%)	0.007
Low	278 (27.4%)	136 (34.5%)	142 (22.8%)	<0.001

a During 12 months observation period.

Prevalence of 30.2 % (n = 307) was found for *Af* colonization within the 12 months of observation period (**Table 1**). *Af* colonization was significantly associated with urban life (**Tables 2**, **4**) including an increased prevalence of 36.6 % of *Af* colonization in large cities (**Table 2**). Binary logistic regression of *Af* colonization was performed including gender (male gender: p = 0.244), age (p < 0.001), different types of residential area (p = 0.022) and population densities (p = 0.918). Adjusted odds ratios for age and residential areas were shown in **Table 4**.

**Table 2:** *Af* colonization and ABPA diagnosis in different types of residential area for all included CF patients.

All included CF patients, n = 1016					
	Large city, n = 314	Urban district, n = 461	Rural district, n = 141	Sparsely populated rural area, n = 100	P value
*Af* colonization, n (%)	115 (36.6 %)	138 (29.9 %)	34 (24.1 %)	20 (20.0%)	0.004
ABPA, n (%)	64 (20.4%)	67 (14.5%)	16 (11.3%)	18 (18%)	0.054
**CF patients >= 18 years, n = 395**					
	**Large city n = 91**	**Urban district n = 186**	**Rural district n = 68**	**Sparsely populated rural area n = 50**	**P value**
*Af* colonization, n (%)	22 (24.2 %)	33 (17.7%)	8 (11.8 %)	10 (20.0%)	0.247
ABPA, n (%)	11 (12.1%)	6 (3.2%)	7 (10.3%)	6 (12.0%)	0.021
**CF patients ≥ 18 years, n = 621**					
	**Large city n = 223**	**Urban district n = 275**	**Rural district n = 73**	**Sparsely populated rural area n = 50**	**P value**
*Af* colonization, n (%)	93 (41.7 %)	105 (38.2 %)	26 (35.6 %)	10 (20.0%)	0.039
ABPA, n (%)	53 (23.8%)	61 (22.2%)	9 (12.3%)	12 (24.0%)	0.210

ABPA was documented in 16.2 % (n = 165) of the CF patients (**Table 1**), with a slightly pronounced documentation in large cities but without statistical significance compared to the other types of residential area (p = 0.054; **Table 2**). The individual associations between ABPA diagnosis and the investigated parameters were also examined using logistic regression (n = 1016; male gender: p = 0.913; residential area: p = 0.067; population density: p = 0.424). Odds ratio (1.021) and 95 % confidence interval (1.009–1.034) could only be determined for age (p = 0.001).

In addition to the residential area, population density was determined for analyzation. It shows that the majority of CF patients live in regions with a medium population density (n = 601; 59.2 %) and only 13.5 % (n = 137) in areas with high population density (**Table 1**). No statistical significance was calculated for *Af* (p = 0.357) nor ABPA (p = 0.871) documentation within the observation period in association with population density (**Table 3**).

**Table 3:** *Af* and ABPA documentation in relation to population density for all included CF patients.

All included CF patients, n = 1016				
	Population density			P value
	High, n = 137	Medium, n = 601	Low, n = 278	
*Af* colonization, n (%)	45 (32.8%)	187 (31.1%)	75 (27.0%)	0.357
ABPA, n (%)	21 (15.3%)	101 (16.8%)	43 (15.5%)	0.871
**CF patients >= 18 years, n = 395**				
	**Population density**			**P value**
	**High n = 46**	**Medium n = 213**	**Low n = 136**	
*Af* colonization, n (%)	10 (21.7%)	42 (19.7%)	21 (15.4%)	0.503
ABPA, n (%)	4 (8.7%)	9 (4.2%)	17 (12.5%)	0.017
**CF patients ≥ 18 years, n = 621**				
	**Population density**			**P value**
	**High n = 91**	**Medium n = 388**	**Low n = 142**	
*Af* colonization, n (%)	35 (38.5%)	145 (37.4%)	54 (38.0%)	0.977
ABPA, n (%)	17 (18.7%)	92 (23.7%)	26 (18.3%)	0.306

Among the 1016 patients with CF included in this analysis, n = 395 (38.9 %) were less than 18 years of age. N = 197 (49.9 %) of them were female. Prevalence of 18.5 % (n = 73) was found for *Af*. ABPA was documented in 7.6 % (n = 30) in this cohort (**Table 1**). Both, *Af* colonization and ABPA diagnosis was significantly lower in CF patients <18 years compared to adult CF patients (p<0.001; **Table 1**). Due to small case numbers for CF patients < 18 years, examination of *Af* colonization and ABPA measures regarding the type of residential area (**Table 2**) and population density (**Table 3**), the results have to be carefully interpreted.

N = 621 (61.1 %) CF patients were ≥ 18 years of age and 47.5 % (n = 295) of them were female. Prevalence for *Aspergillus* was 37.7 % (n = 234; **Table 1**) *Af* colonization was significantly associated with urban life in large cities (p = 0.039; **Table 2**). ABPA was documented in n = 135 (21.7%) of the adult CF patients (**Table 1**). No statistical significance was calculated for *Af* colonization (p = 0.977) nor ABPA (p = 0.306) in association with population density (**Table 3**).

## Discussion

This nationwide questionnaire survey explored the association of aspergillosis documentation in different types of residential area and various population densities. *Af* colonization is significantly associated with urban life in large cities (**Tables 2**, **4**). History of ABPA during the 12 months of observation period was not significantly associated with urban or rural life. ABPA tended to be elevated in large cities and sparsely populated rural areas (**Table 2**). In contrast to residential area types, population density measures showed no significance with regard to aspergillosis (except ABPA data for CF patients < 18 years; p = 0.017; **Table 3**). One could speculate that population density is not the decisive factor. This inconsistency may be related to different data origins (BBSPR vs. DESTATIS). In contrast to residential area types, population density was divided in only three categories (high, medium, low), not reflecting extreme values (e.g. large cities and sparsley populated rural area). Maybe other environmental parameters, better reflected by different residential area types, are more important, e.g. architecture, furnishing or exposure to air pollution including particulate matter. A French study previously described *Af* distribution: Urbanized areas and heterogeneous agricultural areas were mentioned to be positively related to *Af* (Rocchi et al., 2020). Particularly, large cities seem to have negative effects. But also, agricultural air pollutants contribute to human health problems (Aneja et al., 2009). Particulate air pollution was associated with an increased risk of pulmonary exacerbations in CF (Brugha et al., 2018), a decline in lung function (Goss et al., 2004) and can exert proinflammatory and immunomodulatory effects (Reinmuth-Selzle et al., 2017). Our findings may reflect that in urban as well as sparsely populated rural areas synergistic effects of more frequent *Af* acquisition and immunomodulatory effects of air pollution influence the CF patients *Af* outcome. It is postulated that, pollution exposure early in life might associate with an increased risk of early infection to certain microbes (Brugha et al., 2018). Furthermore, the exposure to environmental factors might be even more harmful in children than in adults, because of behavioural and physiological factors (Goldizen et al., 2016). This may partly explain the determined frequencies for ABPA in patients <18 years in the studied residential area types. In future, the majority of people will live in urban areas, spending most of their time indoors (Rydin et al., 2012; Leung and Lee, 2016; Flies et al., 2019). Many issues are connected with this progress and the influence of urban environment associated factors will increase. The association between urban environments and disease is complex and context specific. Urban environments pose potential health risks and offer potential health benefits, such as improved access to health care, education, employment, or infrastructure (Flies et al., 2019). These health care aspects could lead to possible bias in the present work. One could speculate, that more severely affected CF patients tend to move to larger cities to benefit from better medical care. Infections require inhalation of fungal spores from the environment (Harun et al., 2010). *Aspergillus* is among the most common life-threatening airborne opportunistic fungus found indoors (Rocchi et al., 2020). The presence of *Af* in CF sputum is associated with worse respiratory quality of life in CF (Hong et al., 2020) and chronic Af is associated with more frequent pulmonary exacerbation dependent hospitalizations (Hong et al., 2018). CF patient registry data has already been used for risk factor analysis and macrolides, inhaled antibiotics and inhaled corticosteroids were associated with persistent *Af* isolation (Hong et al., 2018). In contrast, this questionnaire survey focused on urban and rural areas or population density items. Therefore, the impact of simultaneous exposure to multiple contaminants (other fungi, bacteria) has not been observed. Some species showed differences in concentrations according to geographic locations and the distribution of fungal spores is not homogeneous in the atmosphere (Rocchi et al., 2020). Regional or geographical patterns as well as different outdoor parameters or indoor characteristics (thermo-isolation, ventilation, architecture) were not considered in this study. Furthermore, no information on the frequency of microbiological examinations in the individual CF centres was available and questionnaire associated bias have to be considered. The possible influence of contact with pets will be discussed in detail in a separate article of this thematic special issue (see “Frequent pet contact as risk factor for allergic bronchopulmonary aspergillosis in cystic fibrosis”). Due to the 12 months observation period, the importance of seasonality on *Af* distribution can be excluded. The aim of the study was to investigate whether urban or rural life could affect the appearance of a colonization with *Af* in the respiratory tract of patients with CF. There is increasing evidence that there are adverse effects associated with the environment in large cities. The understanding of potential risk factors for aspergillosis permits the implementation of patient guidance and preventive measures.

**Table 4:** Adjusted odds ratios for age and residential areas of *Af* colonization.

All included CF patients, n = 1016				
	Odds ratio	95% Confidence Interval	P value
Age (years)	1.028	1.017 – 1.038	<0.001
Residential area	–	–	0.019
Large city (vs sparsely populated rural area)	2.114	1.220 – 3.661	0.008
Urban district (vs sparsely populated rural area)	1.724	1.008 – 2.949	0.047
Rural district (vs sparsely populated rural area)	1.265	0.672 – 2.383	0.466

## Conclusion

Urban live should be considered as possible risk factor for colonization with *Af* in the respiratory tract of patients with CF. These new results may raise the awareness of the influence of environmental factors on patient outcomes and should be included in patient guidance and preventive measures.

## Data Availability Statement

The raw data supporting the conclusions of this article will be made available by the authors, without undue reservation.

## Ethics Statement

The studies involving human participants were reviewed and approved by Ethics Committee of the Charité—Universitätsmedizin Berlin. Written informed consent to participate in this study was provided by the participants’ legal guardian/next of kin.

## Author Contributions

CG, PE, ST, UD, KN, and CS contributed to the conception and design of the study. UD and CG organized the database. KN and CG performed the statistical analysis. CG, PE, ST, UD, KN, and CS wrote the manuscript. All authors contributed to the article and approved the submitted version.

## Funding

This work was supported by the German Federal Ministry of Education and Research (BMBF)—Project InfectControl 2020 (“Art4Fun”, 03ZZ0813E).

## Conflict of Interest

The authors declare that the research was conducted in the absence of any commercial or financial relationships that could be construed as a potential conflict of interest.
